# “*Having to Shift Everything We’ve Learned to the Side*”: Expanding Research Methods Taught in Psychology to Incorporate Qualitative Methods

**DOI:** 10.3389/fpsyg.2016.00688

**Published:** 2016-05-10

**Authors:** Lynne D. Roberts, Emily Castell

**Affiliations:** School of Psychology and Speech Pathology, Curtin University, PerthWA, Australia

**Keywords:** attitudes, undergraduate psychology, qualitative research, research methods, epistemological beliefs

## Abstract

In Australia the tradition of conducting quantitative psychological research within a positivist framework has been challenged, with calls made for the inclusion of the full range of qualitative and quantitative methodologies within the undergraduate psychology curriculum. Despite this, the undergraduate psychology curriculum in most Australian universities retains a strong focus on teaching quantitative research methods. Limited research has examined attitudes toward qualitative research held by undergraduate psychology students taught within a positivist framework, and whether these attitudes are malleable and can be changed through teaching qualitative methodologies. Previous research has suggested that students from strong quantitative backgrounds experience some cognitive dissonance and greater difficulties in learning qualitative methods. In this article we examine 3rd year undergraduate psychology students’ attitudes to qualitative research prior to commencing and upon completion of a qualitative research unit. All students had previously completed two 13 weeks units of study in quantitative research methods. At Time 1, 63 students (84.1% female) completed online surveys comprising attitudinal measures. Key themes to emerge from student comments were that qualitative research was seen as an alternative approach, representing a paradigmatic shift that was construed by some students advantageous for meeting future professional and educative goals. Quantitative measures of attitudes to qualitative research were associated with general attitudes toward research, and psychology-specific epistemological beliefs. Changes in attitudes following completion of the qualitative research methods unit were in the hypothesized direction, but non-significant (small effect sizes). The findings increase our understanding of psychology students’ attitudes toward qualitative research and inform our recommendations for teaching research methods within the undergraduate psychology curriculum.

## Introduction

Qualitative research has a low profile in psychology, accounting for less than 10% of indexed empirical research articles published in psychology journals, with these publications predominantly in interdisciplinary and applied journals (Eagly and Riger, 2014). However, an increasing acceptance of the plurality of research methods (Gergen, 2014) has been accompanied by increased interest in qualitative research in psychology (O’Neill, 2002; Ponterotto, 2002, 2005; Karasz and Singelis, 2009; Demuth, 2015) as evidenced by the introduction of an American Psychological Association journal, *Qualitative Psychology*, specifically catering to presenting qualitative psychology findings (Gergen et al., 2015), and a small number of other journals explicitly encouraging the submission of qualitative papers (e.g., *The Journal of Counseling Psychology*; Haverkamp et al., 2005). Despite this, there remain wide differences in research practices and the knowledge and acceptability of qualitative research across sub-domains of psychology (Eagly and Riger, 2014), with quantitative psychological researchers often unaware of the range of qualitative epistemologies and practices available (Demuth, 2015).

In line with the increased interest in qualitative research amongst psychological researchers, qualitative research is increasingly being taught in undergraduate and postgraduate psychology degrees. For example, it is now mandatory for all undergraduate psychology courses in the United Kingdom to include qualitative research methods in the curriculum (Forrester and Koutsopoulou, 2008), although undergraduate research supervisors continue to report that the limited qualitative methods training provided in the undergraduate degree presents difficulties in supervising qualitative undergraduate dissertations (Wiggins et al., 2016). The tradition of conducting quantitative psychological research within a positivist framework is being challenged, with calls made for the inclusion of the full range of qualitative and quantitative methodologies within the undergraduate psychology curriculum (Mitchell et al., 2007; Breen and Darlaston-Jones, 2010; Wertz, 2014). Some universities, including our own, now teach qualitative and mixed methods research, in addition to quantitative methods, to undergraduate psychology students.

Despite the growth of qualitative methods in psychology and the teaching of qualitative methods in undergraduate and postgraduate psychology degrees, limited research has examined the attitudes toward qualitative research held by psychology students. Rabinowitz and Weseen (1997) explored how the quantitative-qualitative debate was experienced by 20 doctoral students in a social-personality psychology program. Most of the quantitatively oriented students expressed concerns that qualitative research was arbitrary, unscientific and particularly susceptible to researcher bias. These students also reported having difficulties evaluating qualitative studies. Murtonen (2005) examined social science, education and psychology students’ preferences, aversions and appreciation of research methods and their readiness to use them. Students tended to have a dichotic attitude toward qualitative and quantitative research methods that was formed before or at the commencement of their studies. Psychology students’ interest in qualitative methods increased when they experienced difficulties in quantitative research. Mitchell et al. (2007) had three psychology students reflect on their experiences learning qualitative research methods as part of their undergraduate degree. One student acknowledged that they had internalized quantitative standards of research, such as external validity and objectivity, and described qualitative research as daunting as these standards appeared to be in direct opposition to the guiding principles of qualitative research. Students also reported that their exposure to qualitative methods was limited and that they had considerable difficulty obtaining the equipment necessary to conduct qualitative research.

More recently, Povee and Roberts (2014) interviewed 21 Australian psychology students and academics about their attitudes toward qualitative research. Qualitative research was seen by some participants as inherent to psychology, with parallels drawn between conducting qualitative research and practicing as a psychologist. Qualitative research methods were viewed as capturing the lived experience of research participants, reducing power differentials between the researcher and participants. However, qualitative research was viewed as less well respected and legitimate than quantitative methods within the field of psychology. Furthermore, viewing psychology in terms of a quantitative paradigm, participants raised concerns about the subjective nature of qualitative research, susceptibility to researcher bias, lack of rigor, inability to generalize beyond the sample and cast doubts about qualitative researchers’ abilities in learning quantitative methods. Limited exposure to qualitative research methods and perceptions that qualitative research was time consuming and requiring large investments in resources were also identified as barriers to conducting qualitative research.

Many of the negative attitudes toward qualitative attitudes expressed in the literature reviewed may be a function of a lack of familiarity and training in qualitative methods. Despite the increasing prevalence of qualitative research in psychological research and education, limited research has examined how attitudes toward qualitative research change with teaching. Previous research has suggested that students from strong quantitative backgrounds experience some cognitive dissonance and greater difficulties in learning qualitative methods than other students (Kleinman et al., 1997; Cooper et al., 2012), resulting in challenges in learning “against the grain” (Eakin and Mykhalovskiy, 2005; Mitchell et al., 2007). Further, within the field of psychology, continuing resistance to qualitative research in some areas (McMullen, 2002) creates a context when methodological diversity may not be valued. Mitchell et al. (2007) highlight the importance of considering the epistemological beliefs of undergraduate psychology students upon commencing qualitative research education, noting the challenges involved in shifting epistemological beliefs.

Research in this area of attitudes has been hampered by the absence of reliable, validated measure of attitudes. However, the recent development and validation of measures of psychology specific epistemological beliefs (Renken et al., 2015) and attitudes toward qualitative research (Roberts and Povee, 2014; based on the qualitative research by Povee and Roberts, 2014), provide instruments suitable for this purpose.

The current study aims to explore undergraduate psychology students’ attitudes to qualitative research, and how these change following exposure to qualitative methods. The context for the study is what has been described as a “typical academic study department in Australia” (Rees, 2013) where staff engage in research on a wide range of topics using a variety of research methodologies, including qualitative and mixed methods (Rees, 2013). Undergraduate psychology students complete two quantitative research methods units in their 2nd year and a qualitative methods unit and a mixed methods unit in their 3rd year of the degree.

The two research questions driving this research are:

(1) What attitudes do undergraduate psychology students hold toward qualitative research prior to commencing training in qualitative research?
(2) Do students’ attitudes to qualitative research change after instruction in qualitative methods?

The first question is exploratory, designed to examine the relationship between general attitudes toward research, psychology specific epistemological beliefs and attitudes toward qualitative research. For the second research question, we hypothesized that instruction in qualitative methods would change attitudes to each of the four components of attitudes toward qualitative research:

H1. Perceptions of the lack of validity of qualitative research would decrease following instruction in qualitative methods.
H2. Perceptions that qualitative research captured the lived experience would increase following instruction in qualitative methods.
H3. Perceptions that qualitative search were time and resource intensive would increase following instruction in qualitative methods.
H4. Self-perceptions of a qualitative orientation would increase following instruction in qualitative methods.

## Materials and Methods

### Participants

Sixty-three 3rd year undergraduate psychology students (84.1% female; age range 18–55 years) at an Australian university participated in this research. An *a priori* power analysis indicated a minimum sample size of 34 participants was required to have the power to detect a medium effect size change in attitudes.

### Measures

Two online surveys were hosted on Qualtrics.com comprising the following measures:

#### Attitudes Toward Qualitative Research in Psychology (Roberts and Povee, 2014)

This measure consists of 18 items expressing attitudes toward qualitative research in psychology. Participants responded on a 7 point response scale ranging from strongly disagree (1) to strongly agree (7). Exploratory and confirmatory factor analysis indicate four factors underlie the measure: ‘perceived lack of validity’ (example item, “Qualitative research lacks scientific rigor”), ‘capturing the lived experience’ (example item, “Qualitative research can capture the complexity of the social world”), ‘qualitative orientation‘ (example item, “The most interesting findings in psychology are obtained with qualitative methods”), ‘and ‘time and resource intensive’ (example item, “Qualitative research is harder to conduct than quantitative research”). The factors each have acceptable internal reliability as indicated by Cronbach’s alpha: validity (0.82); capturing the lived experience (0.73), qualitative orientation (0.73) and time and resource intensive (0.72; Roberts and Povee, 2014).

#### Attitudes Toward Research Scale (Papanastasiou, 2005; Walker, 2010)

The original Attitudes Toward Research Scale (Papanastasiou, 2005) consisted of 32 items that measure general attitudes toward research. In this study we used the shortened version of the measure derived through confirmatory factor analysis (Walker, 2010) that comprises 18 items loading on three factors: research use (10 items), negative attributes of research (4 items), and positive attributes of research (4 items). Each item is responded to on a seven point scale anchored by strongly disagree (1) to strongly agree (7). Each factor has acceptable internal reliability (Cronbach’s alphas all above 0.8; Walker, 2010).

#### Psychology-Specific Epistemological Beliefs Scale (Renken et al., 2015)

This scale comprises 13 items measuring psychology-specific epistemological beliefs. The items load onto three factors: significance of psychological research (example item “Carefully controlled research is not likely to be useful in solving psychological problems”), subjective nature of psychological knowledge (example item “Psychologists in different eras may use different theories and methods to interpret the same natural phenomenon”), and predictability of human behavior (example item “Psychological research can enable us to anticipate people’s behavior with a high degree of accuracy”). Initial validation of this measure has included confirmatory factor analysis, test–retest reliability (correlations ranging between 0.65 and 0.78) and internal reliability (α = 0.54 to 0.80 for subscales, and 0.75 to 0.82 for overall measure; Renken et al., 2015).

One open–ended question at Time 1 asked “How do you feel about completing a unit in Qualitative Research Methods? Why?”

### Procedure

Following approval from Curtin University Human Research Ethics Committee, students enrolled in a compulsory 3rd year psychology undergraduate unit on Qualitative Methods at Curtin University were invited to take part in this research. Participation was voluntary, with students able to select from this and a range of other studies concurrently running through the School’s research participation pool. The first survey was available for 2 weeks at the beginning of semester. Of the 190 students enrolled in the unit, 63 participated (33% response rate) in the Time 1 survey. In the last week of the semester participating students were emailed a reminder to complete the second survey. Of these, 52 students completed the Time 2 survey. Students who completed both surveys were awarded participation points.

Data was downloaded from Qualtrics into SPSS (v. 20) for analysis. There were 13 missing data points in scale items in the Time 1 survey. Little’s MCAR test indicated this data was missing completely at random (χ^2^ = 75.017, df = 891, *p* = 1.000), and the data points were replaced using Expectation Maximization. This dataset was used for exploring the first research question. There were also 13 missing data points in scale items in the Time 2 survey. Little’s MCAR test indicated this data was missing completely at random (χ^2^ = 0.000, df = 860, *p* = 1.000), and the data points were replaced using Expectation Maximization. The Time 1 and 2 datasets were merged, with 35 cases able to be matched on the user-generated codes. This merged dataset was used for exploring the second research question.

## Results

### Qualitative Results

To examine attitudes undergraduate psychology students hold toward qualitative research, response to the open–ended question were content analyzed.

#### Theme: An ‘Alternative’ Methodology

The theme ‘an alternative approach’ reflects students’ tendency to frame their feelings about completing a unit in qualitative research methods in context of previously learned skills and information on quantitative research methods. A number of students suggested that the undergraduate curriculum had been dominated by quantitative research methods. It would seem that, for a number of students, feelings about undertaking a unit in qualitative research methods were informed by previous experiences in quantitative research methods units:

I am very excited to be learning about Qualitative Research methods. As I feel, that up and until now we have mainly focused on the Quantitative/Positivist aspect of Research through the use of statistics and experimental method.

Students expressed that they were looking forward to the prospect of learning approaches to research that were alternative to those used in quantitative research methods. A number of students articulated feeling apprehensive toward qualitative research methods, for example:

I feel intimidated because the content seems like such a contrast to the past research methods units I’ve completed, and I see it as a challenge.

In approaching the study of qualitative research methods, students appeared to construct qualitative methods as an ‘alternative’ approach to research, one that could be understood in terms of its differences and similarities to the dominant paradigm of quantitative research methods:

It should be interesting to compare what i already know about quantitative methods to qualitative and seeing not only the differences but their similarities.

The implication of this construction, perhaps, is reflected in discourse around the relative value of qualitative methods in contrast to quantitative methods. Perhaps the dominance of quantitative methods in the curriculum constructs an impression that the different research paradigms have a relative value attached to them:

“*…we have had the importance of quantitative methods stressed to us so to oppose those methods and ways of thinking is overwhelming.*”

In considering how they felt about completing a unit in qualitative methods, some students noted that both qualitative and quantitative research methods were valuable, for example, one student noted:

“*…both methods of research can be equally important in Psychology.*”

It would seem that attitudes toward learning about qualitative research methods were inextricably linked with previous learnings from units in quantitative research methods.

#### Theme: A Paradigmatic Shift

The theme ‘a paradigmatic shift’ captures students’ reflections on epistemology in the context of learning about qualitative research methods. A number of students regarded qualitative research methods as demanding a different way of operating than required in previous quantitative research methods units, for example:

I am unsure about this unit. There is just some uncertainties I am yet to understand. Comparing this unit to my previous units, this is very theoretical…

Students’ anticipated that learning about qualitative research methods would involve a level of uncertainty and ambiguity not previously encountered in their quantitative research methods units. For example:

I am a little apprehensive because it seems as if there are many gray areas within qualitative research and some aspects of qualitative research are not clearly defined.

A number of students aligned the shift from learning about quantitative methods to qualitative methods with a departure from focusing on numbers and statistics to exploring meaning and experiences. For example, when asked how they feel about the prospect of undertaking the unit, one student reflected:

“*…I’ve never done anything like it before and nervous as I’m not very good with language/art topics but better with statistics.*”

Students indicated that the differences between quantitative and qualitative research methods reflected inherently different ways of approaching research and dealing with data, tantamount to ‘wrapping’ ones “…*head around a whole new set of ideas.”* While a number of students expressed that they were apprehensive about undertaking a unit in qualitative methods, some students anticipated that qualitative research methods may offer a more intuitive way of approaching research than quantitative research methods:

“*I just hope it makes a bit more real life sense than the stats we have completed so far!!!”*

Some students noted the potential for qualitative research methods to offer depth, richness, and complexity, for example:

I believe that Qualitative Research Methods will allow me to incorporate my understanding of human’s (both the subjects and researchers) complexities into the research, rather than attempting to remove our values, attitudes, and contexts from the experiments.

A number of students suggested that the emphasis on exploration, meaning, depth, and complexity inherent to qualitative research methods aligned with their personal interests, “*Excited to learn more about qualitative methods as that what I find interesting- opinions and beliefs people hold and the reasons behind them.*” and with what they understand as the broader aims within the discipline of psychology:

It seems to tie in well with what I had in mind when I signed up to study Psych. I like that we have done quantitative methods first, it seems to ground this unit nicely in the realm of science.

Some students reflected on how they had been socialized to a particular approach to research methods, and anticipated that the alternative approach offered by qualitative research methods may pose a challenge to the dominant epistemological position fostered by previous units of study:

“*It allows us to challenge our thinking and introduces different epistemological ideas/ theories. It will be exciting to see how our views get challenged over this year…*”

#### Theme: Reconciling the ‘Known’ and ‘Uncertain’

The theme captures a key tension emerging from students’ feelings toward undertaking a unit in qualitative research methods. Students often reflected on their feelings toward undertaking the unit in terms of overarching goals. For example, some students expressed that undertaking the unit would be valuable for their future studies (e.g., “*I am very excited to begin qualitative research methods as I hope to be running this sort of research myself in future*”) and careers in psychology “*I feel like it is important to complete this unit as it will assist in my future career in psychology.*”

For these students, learning about qualitative research methods seemed to be constructed as advantageous for meeting future professional and educative goals. While some students reflected on the unit as an opportunity to learn new information and enhance career goals, other students emphasized that completing the unit represented a necessary step in completing their degree. For example:

I’m not excited about completing the unit. It does not interest or stimulate me. However, I know it has to be done in order for me to get the most out of my degree and understand all elements and processes involved in psychological research.

Some students expressed indifference toward the content of the unit, expressing an eagerness to complete the unit and engage in future professional work:

I see the unit as a means to an end, the means to complete my degree in psychology and begin working in the field.

A number of students reflected on their feelings toward undertaking the unit in terms of how they felt this might impact upon their academic performance. Some students expressed apprehension toward the unit based on performance in previous research methods units. For example, those students who felt as though they had experienced difficulty in previous research methods units questioned their ability to perform well in qualitative research methods:

Initially I felt very distressed at the thought of completing this unit. Having struggled with previous Psychological Science units, I was anxious as I was unsure if I would find the unit more difficult and therefore, perhaps not pass it.

Other students expressed that the novelty of qualitative research method may pose a particular threat to academic success:

“*I’m intrigued to find out what it’s all about. I’m interested in learning a new way of approaching research questions, and a new way of thinking about knowledge and understanding, in general. I am nervous about the assignments and this unit, as its way outside my wheelhouse – I hope I don’t bomb out and ruin my average and all the hard work I’ve put in so far.*”

For students who had experienced quantitative research methods as challenging, qualitative research methods offered an opportunity to learn a different approach which may offer an opportunity to perform:

The reason why I am interested in this unit is due to the fact that it is different to Quantitative research. In which using the computer for numbers was quiet confusing and slightly harder to grasp.

For some, the opportunity to embrace the uncertainty and novelty of qualitative research methods was appreciated, for others, the idea of undertaking a unit in qualitative research methods was seen as posing a challenge to academic performance, and potentially undermining their ability to do well in their studies.

### Quantitative Results

Our first research questions asked what attitudes undergraduate psychology students hold toward qualitative research prior to commencing training in qualitative research. The scale scores and scale reliabilities from the Time 1 survey are presented in **Table 1.** On average, incoming students agreed that qualitative research captured the lived experience of participants and was time and resource intensive; however, they did not agree that qualitative research lacked validity or that they were qualitatively oriented.

**Table 1:** Descriptive statistics for scale measures Time 1 (*N* = 73).

Measure	Range	Mean (*SD*)	Alpha
**Attitudes toward qualitative research**			
Perceived lack of validity	1.00–7.00	3.22 (1.38)	0.90
Capturing the lived experience	2.00–7.00	5.85 (1.09)	0.96
Time and resource intensive	1.50–6.25	4.33 (1.06)	0.76
Qualitative orientation	1.00–6.25	3.76 (1.05)	0.60
**Attitudes toward Research**			
Negative attributes of research	1.75–6.50	3.89 (1.17)	0.78
Positive attributes of research	1.75–7.00	4.62 (1.08)	0.74
Research use	3.40–7.00	5.70 (0.87)	0.89
**Psychology specific epistemological beliefs**			
Significance of psychological research	1.00–4.80	2.60 (0.90)	0.74
Subjective nature of psychological knowledge	3.60–6.80	5.38 (0.80)	0.79
Predictability of human behavior	3.33–7.00	4.87 (0.76)	0.66


To examine the first research question, scale measures of attitudes to qualitative research were correlated with measures of general attitudes toward research and psychology specific epistemological beliefs (see **Table 2**). Key findings in relation to general attitudes toward psychological research were that positive attitudes toward research were negatively associated with a qualitative orientation, and both positive attitudes and perceptions of research usefulness to the profession were positively correlated with viewing qualitative research as capturing the lived experience. Epistemological beliefs were also associated with attitudes toward qualitative research. In particular, perceptions of the subjective nature of psychological knowledge were positively associated with viewing qualitative research as capturing the lived experience.

**Table 2:** Relationships between psychology specific epistemological beliefs, attitudes toward research and attitudes toward qualitative research (*N* = 63).

	Attitudes to qualitative research			
	
Measure	Lack of validity	Lived experience	Resource intensive	Qualitative orientation
**Attitudes to research**				
Negative	0.085	0.097	0.157	0.244
Positive	0.192	0.248^∗^	0.212	-0.327^∗∗^
Research use	0.169	0.355^∗^	0.230	-0.136
**Epistemological beliefs**				
Significant	0.038	-0.194	-0.091	0.221
Subjective	-0.107	0.511^∗∗^	0.078	0.027
Deterministic	-0.104	0.203	0.249^∗^	-0.015


*^∗^*p* < 0.05, ^∗∗^*p* < 0.01, 2-tailed.*

To examine the second research question, four repeated measures *t*-tests were conducted on the four subscales of the *Attitudes toward Qualitative Research in Psychology* measure to test the four hypotheses. The mean scores and standard deviation for each scale at each time point are presented in **Table 3.** While all findings were in the hypothesized direction, there were no significant differences between Time 1 and Time 2 scores on the measures. Using Cohen’s conventions, the effect sizes for each subscale except ‘Time and Resource Intensive’ were small.

**Table 3:** Pre and post-test scores on attitudes toward qualitative research scale (*N* = 35).

	Pre-test Mean (SD)	Post-test Mean (SD)	Effect size Cohen’s *d*
Perceived lack of validity	3.29 (1.48)	3.00 (1.22)	0.22
Capturing the lived experience	5.88 (0.85)	6.09 (0.88)	0.25
Time and resource intensive	4.19 (1.09)	4.23 (0.98	0.04
Qualitative orientation	3.78 (1.11)	4.10 (1.28)	0.27


## Discussion

The aim of our research was to explore undergraduate psychology students’ attitudes to qualitative research, and how these change following exposure to training in qualitative research methods. Prior to commencing training in qualitative research, we found students expressing mixed attitudes toward studying qualitative research methods. Based on the mean score for the ‘qualitative orientation’ subscale falling slightly below the mid-point of the sub-scale, on average students perceived themselves more strongly quantitatively than qualitatively oriented. The qualitative findings indicate that students viewed qualitative research methods as something ‘other’ than, and in opposition to, the quantitative research methods that they had been taught to date, with some students apprehensive about the prospect of learning a new methodological approach. These attitudes, represented in the theme ‘an alternative methodology,’ underpin a world view that qualitative and quantitative methods are dichotomous.

Viewing qualitative and quantitative methodologies as in opposition with each other is also reflected in the theme, ‘a paradigmatic shift,’ where student comments indicated that qualitative research was seen as more complex and interpretative than positivist quantitative methodologies, requiring a different way of thinking. The quantitative results indicate that epistemological beliefs about the subjective nature of psychological knowledge were strongly positively associated with the attitude that qualitative research captured the lived experience.

The third theme, ‘reconciling the ‘known’ and ‘uncertain,” represents the goal-oriented views expressed by some students. Completing qualitative methods training was seen as beneficial to future studies, to completing their degree and to future work. Deterministic beliefs about the predictability of human behavior were associated with beliefs that qualitative research was (unnecessarily) time and resource intensive, presumably in comparison to quantitative research. The uncertainty about qualitative methods is also captured in the greater variance in scores on the ‘perceived lack of validity’ subscale of the Attitudes to Qualitative research measure, in comparison to other subscales of the same measure, indicating the greater divergence of views about the (in)validity of qualitative research.

The dominance of quantitative methods in the first 2 years of the undergraduate curriculum constructs a tension for students when approaching qualitative research methods. While students expressed that they were looking forward to learning what qualitative approaches to research could offer beyond those advantages offered by quantitative methods, for some, undertaking qualitative research methods posed a threat to their performance and prior learnings. The teaching of quantitative methods prior to qualitative methods sets quantitative methods up as the main-stream, preferred research orientation in psychology. This focus on quantitative methodologies, often with limited reference to the underlying epistemological values, positions qualitative research as the alternative, and ‘lesser’ methodological paradigm.

The privileging of quantitative methods in undergraduate psychology education may be in contrast to the expectations of students electing to study psychology. On average, undergraduate psychology students have greater interest in practitioner than research activities (Holmes and Beins, 2009; Holmes, 2014). In summarizing the literature on teaching introductory research methods Earley (2014) noted that across disciplines (including psychology) students have misconceptions about research, see little relevance of research methods to their planned future careers, and may lack interest and motivation. The high mean score on the ‘capturing the lived experience scale’ of the Attitudes Toward Qualitative Research measure in this research suggests that despite socialization into psychology as a quantitative science, many students continue to see value in qualitative approaches, even after quantitative research training.

When reoriented to qualitative methods in the 3rd year of their undergraduate psychology degree, students may experience some dissonance between their (post)positivist quantitative methods training with the emphasis on control, rigor and generalisability and the competing values (including the embracing of exploration, subjectivity, and experience) associated with qualitative methods and their conceptions of psychology upon entering the degree. Students identified that there is a level of uncertainty and ambiguity in qualitative research that they had not previously encountered in quantitative research units, that the qualitative approach may challenge them and require some shifts in thinking. Students may initially struggle with holding multiple ways of knowing simultaneously in mind, and how to integrate their thinking about these. This process occurs in the context of students ultimately striving to ‘perform’ and achieve good grades, potentially restricting ‘deep’ or meaningful learning where they embrace the unknown and the risk of ‘getting it wrong.’ These findings are consistent with previous findings indicating that students from strong quantitative backgrounds experience dissonance when faced with qualitative approaches (Kleinman et al., 1997; Cooper et al., 2012). Integrating qualitative research methods against a backdrop of what they have learned about quantitative methods, qualitative methods inevitably become the ‘alternative’ to quantitative methods.

We were interested in whether students’ attitudes to qualitative research changed after instruction in qualitative methods. We found that completion of the qualitative research methods unit resulted in small increases in attitudes toward qualitative research in the hypothesized direction, but these shifts were not statistically significant. It is possible that attitudes toward research are already largely ‘set’ following socialization into psychology as a (quantitative) science and are resistant to change. Difficulties in in learning “against the grain” have been reported previously (Eakin and Mykhalovskiy, 2005; Mitchell et al., 2007). Further, increasing knowledge of research methods does not necessarily result in increased positive attitudes (Sizemore and Lewandowski, 2009). However, the current research was limited by the small sample size, high attrition rates and difficulties in matching pre- and post- responses. Being asked to take part in study may also have contributed to the perception that qualitative methods are different, an inevitable aspect of research in this area. Further research using larger samples is required to more fully test the malleability of psychology students’ attitudes toward qualitative research through education. Individual case studies may provide insights into how student perceptions change through experiencing qualitative research training. Those prospective challenges identified by students were based on anticipation, as opposed to engagement with, and reflection upon, experiences with qualitative research methods training. Exploring students’ reflections on their engagement in qualitative research methods training after-the-fact may give further insight into the difficulties encountered in in practice.

The ordering of teaching quantitative and qualitative research may be an important consideration in shaping students’ orientation toward the full range of research methods. The current focus on teaching quantitative research before qualitative research privileges quantitative research and sets qualitative research as the ‘alternative’ methodology. This curriculum structure is perhaps not conducive to fostering in students an appreciation of the methodological diversity (McMullen, 2002) which is valued in contemporary approaches to psychological research. Embedding teaching of the epistemological foundations of psychology (Breen and Darlaston-Jones, 2010) and the full range of methods and methodologies available from the start of the undergraduate psychology degree may help to legitimize qualitative findings and position qualitative research as valued within psychology (Gough and Lyons, 2016). It may also serve to remove the false dichotomy between qualitative and quantitative methods and lay the foundations for future mixed methods research.

## Author Contributions

LR had overall responsibility for the design and conduct of the research, writing the literature review and analyzing the quantitative data. EC conducted the analysis of qualitative data. Both authors contributed to the writing of the manuscript.

## Conflict of Interest Statement

The authors declare that the research was conducted in the absence of any commercial or financial relationships that could be construed as a potential conflict of interest.
